# Supplementary figures and images for: Effect of glycogen synthase kinase-3 inactivation on mouse mammary gland development and oncogenesis
Source: Oncogene. 2014 Sep 8;34(27):3514–26. doi: 10.1038/onc.2014.279 (PMC4490903; doi:10.1038/onc.2014.279)

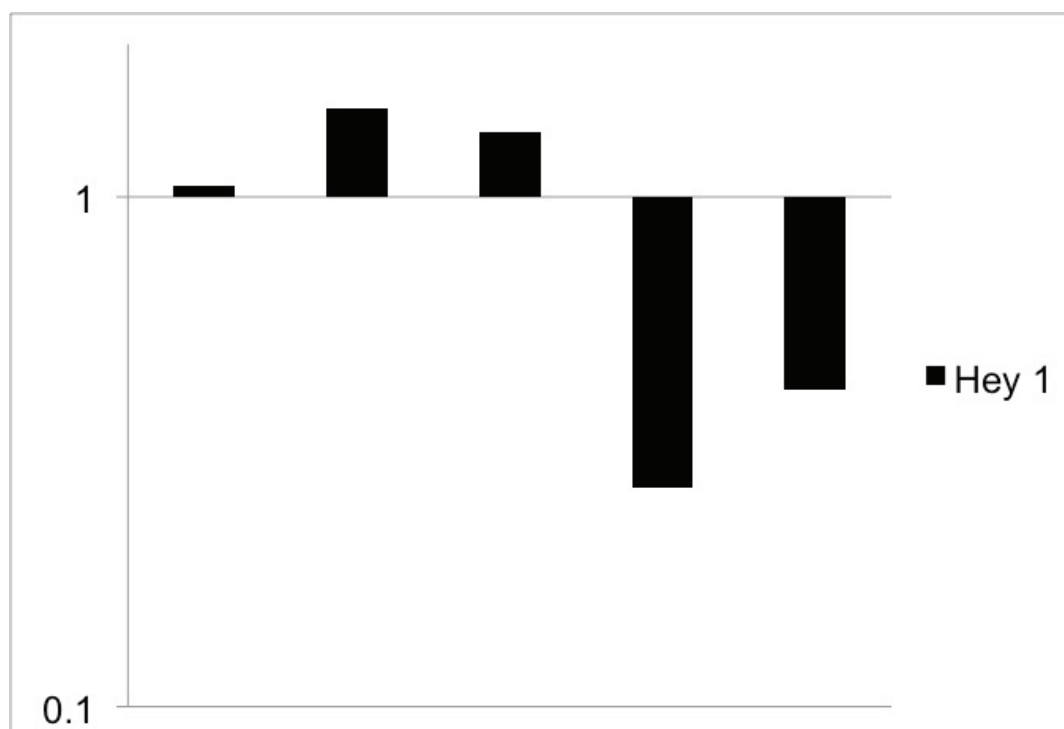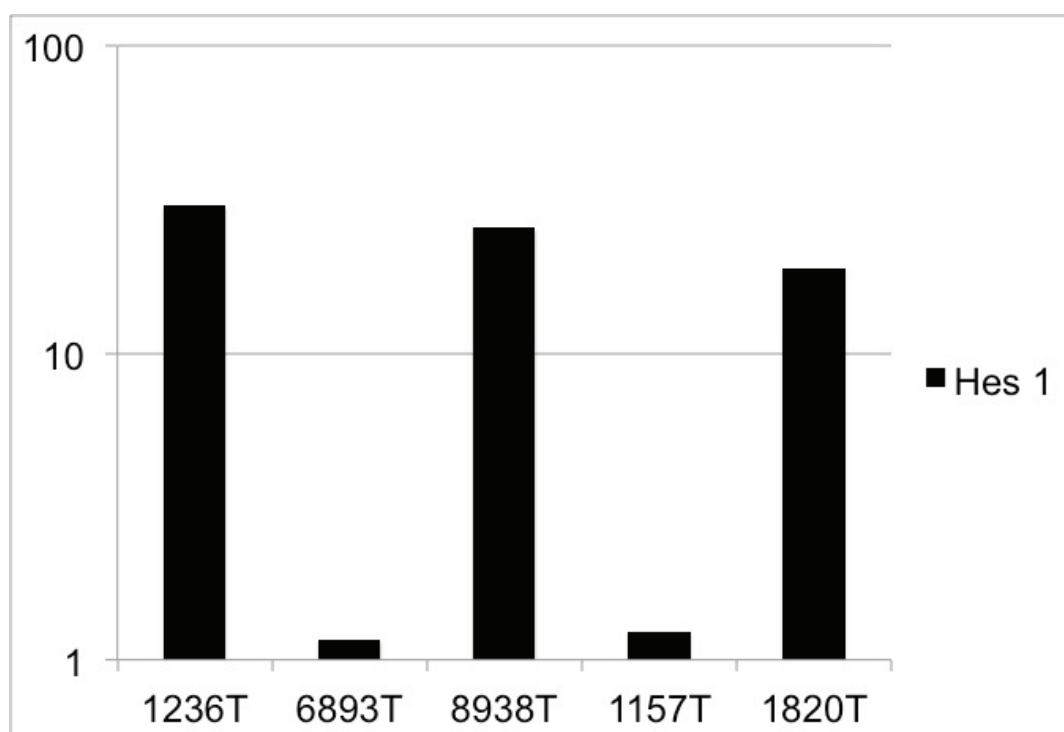

Supplement: Supplementary Figure S1 [file onc2014279x1.pdf]

A

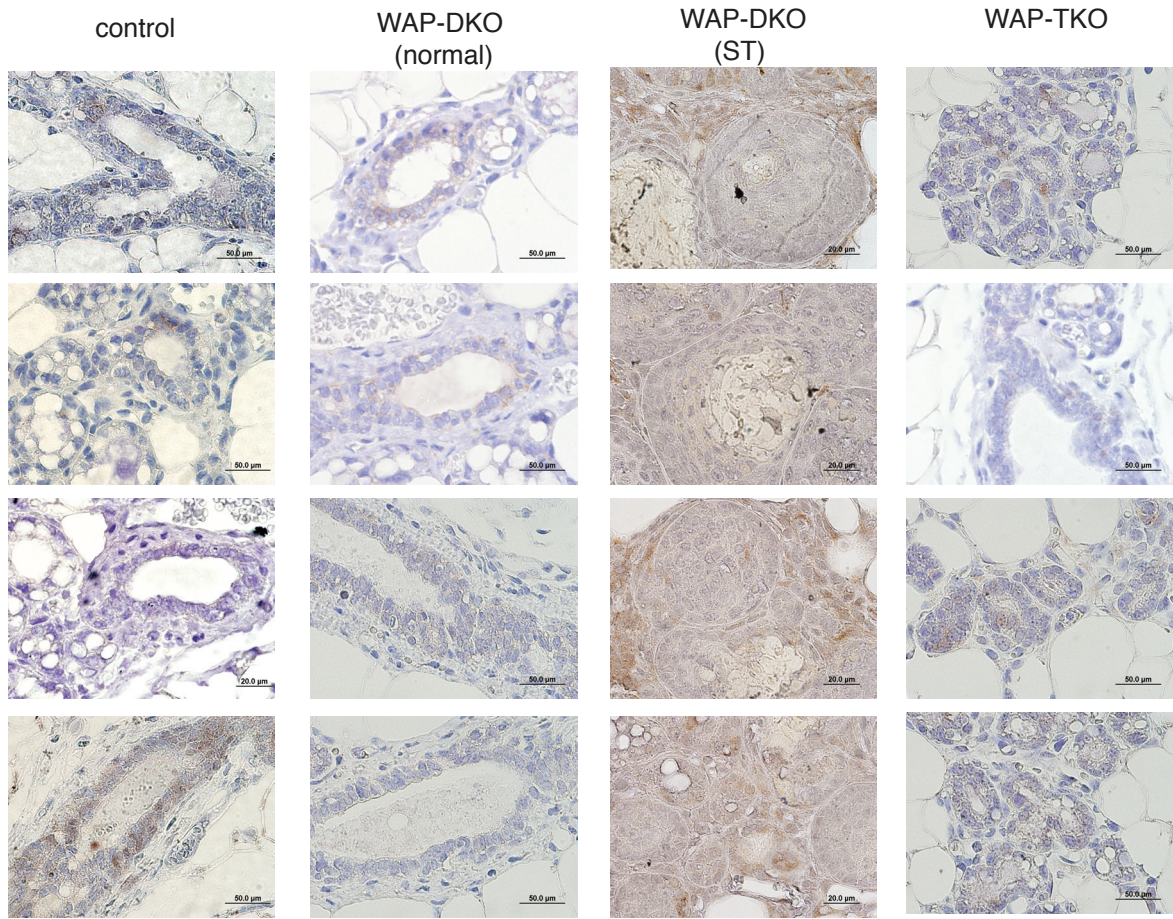

B

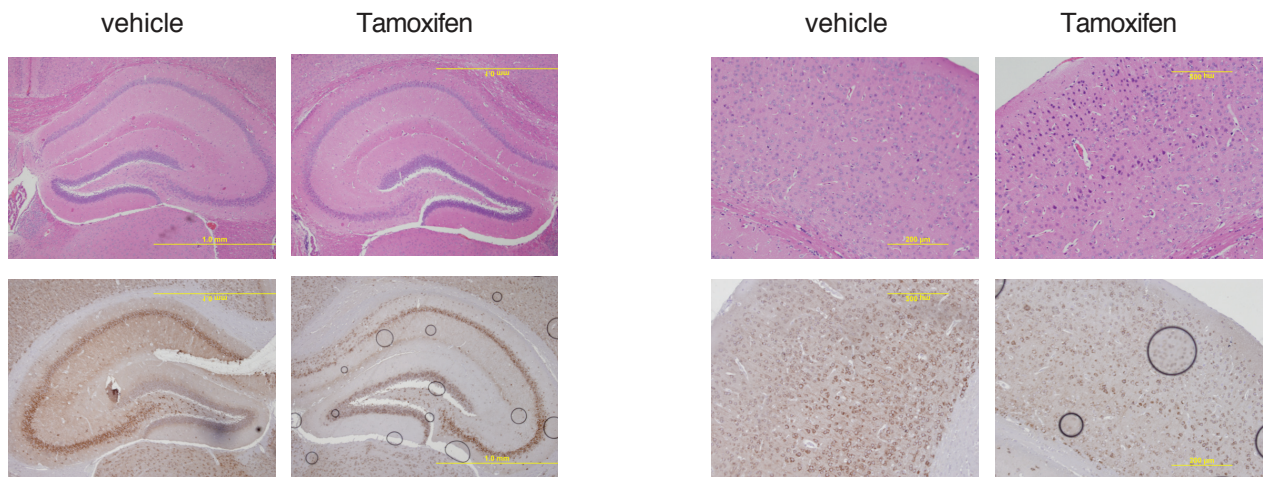

Supplement: Supplementary Figure S2 [file onc2014279x2.pdf]
