# Supplementary material for: Effect of glycogen synthase kinase-3 inactivation on mouse mammary gland development and oncogenesis
Source: Oncogene. 2014 Sep 8;34(27):3514–26. doi: 10.1038/onc.2014.279 (PMC4490903; doi:10.1038/onc.2014.279)
Supplement: Supplementary Information [file onc2014279x3.doc]

We thank the reviewers for their perspective and insightful critiques, which we address below.

Responses to Reviewer #1

**Figure 2, β-catenin levels and localization**

Close inspection of mid-pregnant glands from WAP-DKO and WAP-β-catenin^Active^ stained show both nuclear and cytoplasmic β-catenin, indicative of active Wnt signaling whereas only membranous localization is observed in control tissues. As the reviewer suggested, we have now included magnified insets in manuscript figures to better show this observation. Quantitation of β-catenin is difficult to attain, especially using tissue immunostaining. Although gene dosage studies have been attempted, β-catenin reporter assays and Axin RNA levels were measured only in MEFs (1, 2). These studies suggest that specific levels of canonical Wnt signaling are required to expand the mammary stem/progenitor cell compartment and result in mixed lineage (metaplastic) tumors. Thus, epidermoid transdifferentiation and no significant difference between tumor onset and histopathology between DKOs and β-catenin^Active^ are strong indicators that phenotypes observed in DKOs are largely attributable to activation of β-catenin.

**Effect of GSK-3 and β-catenin on CD24:CD49f stem cell profile**

β-catenin^Active^ mammary epithelial cells (MECs) were sorted into CD24^hi^ and CD49f^hi^ fractions with only the CD49f^hi^ population capable of forming secondary mammospheres (MS) although CD24^hi^ cells were regenerated. This point has now been clarified in the manuscript. Comparing β-catenin activity level within the CD24^hi^ and CD49f^hi^ MECs as well as in MECs from GSK-3 DKOs might further address the question of mechanistic causation of GSK-3 DKO phenotypes but the reliability of the ABC has been questioned (3) and double deletion of GSK-3 yields maximally active β-catenin in other cell types (4).

**3. Effect of GSK-3 loss on developmental pathways**

Despite testing several Notch Intracellular Domain (NICD) antibodies from various commercial sources, we were unable to reliably detect an expected signal in mammary gland samples. qRT-PCR analysis of whole WAP-TKO tumor mRNA evaluating Notch target genes Hes1 and Hey1, revealed more than 10-fold increase in Hes1 expression in 3 of 5 tumors while Hey1 was only modestly elevated (<2-fold) in 3 of 5 tumors and reduced in 2 cases (Supplementary Fig S1 for reviewers). These inconsistencies likely reflect tumor heterogeneity as only random slices of each tumor were tested.

We anticipate that effects of GSK-3 on Wnt, Notch and Hedgehog signaling are ongoing however, different portions of the total GSK-3 pool may participate in the regulation of these pathways. GSK-3 localizes to different cellular compartments and may be partially sequestered in multi-vesicular bodies (MVB) (5). Hence, distribution of GSK-3 may be dependent on cellular context and the events integrated by GSK-3 and the relative strength of their output will determine observed cellular responses. Whereas WAP-TKO tumors showed significant activation of Hh and γ-catenin/plakoglobin, these pathways were essentially unchanged in WAP-DKO tumors suggesting GSK-3 predominantly functions within the β-catenin destruction complex to regulate the Wnt pathway when β-catenin is intact. We have included an earlier remark on induction of γ-catenin/plakoglobin in the Results section (page 14).

Response to Reviewer #2

“The figures and figure legends are hard to follow and do not adequately allow the work to be assessed. They also do not link well with the main text. This is particularly the case for Figs 1-4.”

We believe we have improved the description of the figures (see below for specific changes) and linkage to text.

**“**A number of alternative interpretations of aspects of the data might be possible. For example, could cre-deletion of 4/4 GSK-3 alleles enhance cell death leading to the selection of cells that have lost 3/4 alleles? Do epithelial cells lacking GSK-3 enhance the proliferation of neighbouring normal cells?”

Based on prior findings, we think it unlikely that loss of all GSK-3 alleles results in cell death and we do not believe that data support the hypothesis of selective pressures for generation of ¾ GSK-3 KO for the following reasons:

a) PCR genotyping of tail DNA definitively distinguishes between double flox and single alleles of GSK-3β, thus in GSK-β^FL/FL^ cells where WAP-Cre is expressed we expect recombination to take place with similar frequencies at all 4 LoxP sites, barring any mutations within the core and/or flanking inverted repeats or LoxP-site location-dependent effects due to local DNA structure. Such instances are extremely rare and no such events have been observed in other tissues (liver, T-cells, brain, muscle) where GSK-β^FL/FL^ excision was carried out using different Cre drivers in our and other laboratories, e.g. (6, 7) (8) and unpublished.

b). PCR genotyping of purified primary mammary epithelial cells (MECs) clearly shows the expected flox-deleted band together with absence of any remaining floxed alleles of GSK-β following infection with Ad-Cre-GFP. GSK-3 DKO MECs not only resulted in efficient generation of mammospheres (MS) but also contributed to regeneration of the squamous phenotype when injected into cleared fat pads (Fig 1D of manuscript) in a cell-autonomous manner. GSK-3 DKO cells could be transplanted into wild-type glands to determine any effects on proliferation of neighboring cells. Although this precise experiment was not conducted, we did inject WAP-DKO tumor MECs and found neither tumor formation nor any effects on the existing morphology or function of the endogenous glands suggesting loss of GSK-3 has no observable paracrine effects.

c). Data from both published and unpublished work by ourselves and others consistently show loss of all 4 GSK-3 alleles is required and responsible for the most overt phenotypes, including tumor formation, across different tissues and ¾ KO mice are often used for controls for the compete knockout.

“There are a number of 'obvious experiments' that are missing. Perhaps there are reasons why these failed? If so, it would be helpful if these were discussed, together with the restrictions that their absence places on the interpretation of the data. For example, the lack of cell-by-cell GSK-3b staining in IHC or FACS prevents interpretation of gene deletion frequencies etc.”

The level of GSK-3β expression analyzed via immunohistochemistry (IHC) in pregnant wild-type mammary glands was found to be low and variable making comparison with WAP-DKO difficult (see Supplementary Fig S2A for reviewers). The same GSK-3β antibody (CST 9315) confirmed loss of expression of GSK-3β in brain sections of mice carrying the same floxed allele of GSK-3β where excision was induced by a Tamoxifen-regulated brain-specific Cre (Supplementary Fig S2B). The WAP-Cre transgene is known for non-uniform expression within the mammary gland and was demonstrated to be limited to 15% of the CD24^+^ MEC fraction in 6-month old, multiparous mice of mixed genetic background (9). This variability was confirmed via Cre IHC staining in Fig. 2 of the manuscript where the representative sections indicate Cre^+^ cells constitute only a proportion of the epithelium in mid-pregnant WAP-DKO and WAP-β-catenin^Active^ glands, a time point when WAP is most highly expressed (10-12). It is for these reasons we chose to show the more sensitive PCR genotyping data to confirm excision in mid-pregnant glands while immunoblotting of tumors and adjacent normal glands (which are largely comprised of squamous epithelium) prominently demonstrates near complete loss of the GSK-3β allele in WAP-DKOs.

For the above-mentioned reasons primarily relating to GSK-3β expression, all evidence collected shows that 4 alleles of GSK-3 must be lost to initiate mammary tumor formation. Indeed, the rare incidence of tumors in ¾ WAP-GSK-3 KO and their extended latency and distinct histopathology suggest these may represent a background level of spontaneous mammary tumor formation previously reported in the FVB strain as this level was also observed in our older mouse colony controls (13) (14).

“Data has been shown for the effect of WAP-TKO on the activation levels of Hh and Notch pathways - were these studies carried out in parallel on WAP-DKOs? Would be interesting to look at this, but not strictly needed here.”

**Hh and Notch activation in WAP-DKO glands**

These experiments are interesting but were not performed. However qRT-PCR analysis of Notch target genes Hes1 and Hey1 showed no elevation of these mRNA transcript levels in WAP-DKO tumors and, similarly, no Gli1 upregulation was observed in immunoblots suggesting neither pathway contributes significantly to tumor development in absence of GSK-3 alone.

The clarity and details of figure legends, figure labelling and in text referencing of figures requires improvement.

• (Fig. 1A) Labeling of lanes is essential. It is unclear what genotypes were examined and what PCRs were carried out in each case. Also, what size are the products? All this is important since it can help address questions such as ' Do all 4 alleles need to be lost for tumor formation?', 'What is the proportion of cells that have lost GSK-3b?' There also appears to be a mismatch between the main text and the figure. In the text it states that WAP-DKO virgin and pregnant samples are analysed. Yet the figure legend only refers to pregnant tissue. This difference is important since the levels of deletion are much greater from the WAP transgene in pregnancy. At present, it is impossible to review the paper adequately as this information is missing.

Fig 1A: Lane labels and PCR product size within figures and legends are now included in a revised figure.

• (Fig. 1B.) Not particularly clear what +-+ indicate. Are there 2 repeats?

Fig 1B: Panels are now described in greater detail. + and + represent two different Cre+ animals.

• Fig.1C. Why is there no GSK-3b immunostaining? Please define 'ghost cells' and identify in the sections. Conclusion in text about where cells arose from. Is this possible to assert from sections?

Fig 1C: Addressed above. Labels within figures and legends are now included. Ghost cells are identified by asterix. Cell origin sentence has been deleted (page 8). These were conclusions from pathological examination (by H.A.).

• Fig.1D Hard to follow order of figures - this seems to be much later in paper.

Fig 1D: The order of text has been changed.

• Figure 3. What is the fluorescent image? GFP? What proportion of cells are GFP+? Was GFP positivity part of the gating parameters? Does the proportion of GFP+ cells change over time with passage? There is an 8-fold increase in ++ positive cell numbers (stem) in the GSK-3 nulls. This is not 'unchanged' as stated in the main text. It may be less than that observed in the b-catenin mutant, but could this be because double mutant cells die? A way to check this would be to stain by FACS for GSK-3 expression.

Fig 3: Labels within figures and legends are now included.

The proportion of GFP^+^ cells declines over the course of the assay but is very close to 100% positivity at 2 days post-infection. Since Cre expression is coupled to GFP, initially nearly all cells are expected to express Cre resulting in recombination at the GSK-3β or β-catenin LoxP loci. Over time, while GFP positivity is diluted out of the population gene excision is propagated at each cell division. Hence, GFP positivity is not expected to reliably reflect the proportion of deleted cells at final analysis conducted at day 12 post-infection. Indeed, when a GFP gate was applied, low GFP^+^ cell counts precluded statistical analysis and all subsequent analysis was performed without gating for GFP.

The PCR genotyping shown was performed on an aliquot of each analyzed population, reflecting persistence of efficient excision throughout the experiment.

• Fig4A. Why are bands at different sizes in the GSK-3 mutant? - unclear that there are lower levels of GSK-3b. There appears to be more GSK-3β in the tumor than normal tissues, contrary to the conclusion drawn that GSK-3β has been lost.

• (Fig. 4B, C) Legends do not mention the 3/4 WAP-DKO condition featured in the figures, but instead cite WAP-TKO.

• "Following a median 6-7 months... atypical squamous transdifferentiation (Table 1, Fig 4C)." Figure 4B is the more appropriate figure here.

Fig 4A

There is a slight shift in the GSK-3β band in the normal, adjacent sample but this is most likely an artifact of the gel or sample as this not consistent across all glands and tumors examined. ‘Adjacent normal’ glands of tumor-bearing animals often contain squamous transdifferentiation and hence are expected to be depleted of GSK-3β. Both tumor and normal/adjacent samples contain non-epithelial cell types (& see above re: incompletion of WAP deletion). Hence, although reduced, residual expression of GSK-3β is expected in such samples. The elevated levels of β-catenin in the tumors is a gain-of-function effect and hence can be more easily observed over the background.

Fig 4B, C: This was an error. The labels now correctly reflect the specific transgenics used.

• Fig6B - variation in GSK-3b levels in control animals. How many animals were examined in experimental. Are differences significant?

We have added further information and clarifications to the legend of figure 6.

**Legends for the two supplementary figures for the reviewers.**

**Figure S1: Activation of Notch pathway in WAP-TKO tumors.** mRNA transcript levels of Notch target genes, Hes1 and Hey1. RNA was isolated from random slices of different tumor tissues, reverse-transcribed to generate cDNA templates using SuperScriptIII First-Strand Synthesis System (Life Technologies) that were subjected to qRT-PCR with appropriate primers.

**Figure S2: GSK-3β expression in control and transgenic tissues. (A)** Mammary tissues from mid-pregnant control, WAP-DKO or WAP-TKO mice were stained for GSK-3β as described in the manuscript. Sections from normal glands and those with squamous transdifferentiation (ST) are shown. **(B)** Paraffin-embedded brain sections of CamkIIα-Cre^ERT2^-GSK-3α^FL/FL^; GSK-3β^FL/FL^ mice treated with vehicle or Tamoxifen (as indicated) were subjected to H&E (upper panels) or GSK-3β (bottom panels) staining as described in the manuscript. For activation of CamkIIα-Cre^ERT2^ promoter, 5mg of Tamoxifen (Sigma, Oakville, Canada) was orally administered to 8-week old mice, positive or negative for CamkIIα-Cre^ERT2^ for 5 consecutive days. Tamoxifen was dissolved in 90% sunflower seed oil and 10% ethanol to a final concentration of 50mg/ml (100μL/mouse/day). Brain tissues were harvested 4-6 weeks following last Tamoxifen treatment.

**References**

1.	Bakker ER, Hoekstra E, Franken PF, Helvensteijn W, van Deurzen CH, van Veelen W, et al. beta-Catenin signaling dosage dictates tissue-specific tumor predisposition in Apc-driven cancer. Oncogene. 2013;32(38):4579-85.

2.	Gaspar C, Franken P, Molenaar L, Breukel C, van der Valk M, Smits R, et al. A targeted constitutive mutation in the APC tumor suppressor gene underlies mammary but not intestinal tumorigenesis. PLoS genetics. 2009;5(7):e1000547.

3.	Maher MT, Flozak AS, Hartsell AM, Russell S, Beri R, Peled ON, et al. Issues associated with assessing nuclear localization of N-terminally unphosphorylated beta-catenin with monoclonal antibody 8E7. Biology direct. 2009;4:5.

4.	Doble BW, Patel S, Wood GA, Kockeritz LK, Woodgett JR. Functional redundancy of GSK-3alpha and GSK-3beta in Wnt/beta-catenin signaling shown by using an allelic series of embryonic stem cell lines. Developmental cell. 2007;12(6):957-71.

5.	Taelman VF, Dobrowolski R, Plouhinec JL, Fuentealba LC, Vorwald PP, Gumper I, et al. Wnt signaling requires sequestration of glycogen synthase kinase 3 inside multivesicular endosomes. Cell. 2010;143(7):1136-48.

6.	Kim WY, Wang X, Wu Y, Doble BW, Patel S, Woodgett JR, et al. GSK-3 is a master regulator of neural progenitor homeostasis. Nature neuroscience. 2009;12(11):1390-7.

7.	Kim YC, Clark RJ, Pelegri F, Alexander CM. Wnt4 is not sufficient to induce lobuloalveolar mammary development. BMC developmental biology. 2009;9:55.

8.	Radulescu S, Ridgway RA, Cordero J, Athineos D, Salgueiro P, Poulsom R, et al. Acute WNT signalling activation perturbs differentiation within the adult stomach and rapidly leads to tumour formation. Oncogene. 2013;32(16):2048-57.

9.	Jiang Z, Deng T, Jones R, Li H, Herschkowitz JI, Liu JC, et al. Rb deletion in mouse mammary progenitors induces luminal-B or basal-like/EMT tumor subtypes depending on p53 status. The Journal of clinical investigation. 2010;120(9):3296-309.

10.	Robinson GW, McKnight RA, Smith GH, Hennighausen L. Mammary epithelial cells undergo secretory differentiation in cycling virgins but require pregnancy for the establishment of terminal differentiation. Development. 1995;121(7):2079-90.

11.	Wagner KU, Boulanger CA, Henry MD, Sgagias M, Hennighausen L, Smith GH. An adjunct mammary epithelial cell population in parous females: its role in functional adaptation and tissue renewal. Development. 2002;129(6):1377-86.

12.	Booth BW, Boulanger CA, Smith GH. Alveolar progenitor cells develop in mouse mammary glands independent of pregnancy and lactation. Journal of cellular physiology. 2007;212(3):729-36.

13.	Nieto AI, Shyamala G, Galvez JJ, Thordarson G, Wakefield LM, Cardiff RD. Persistent mammary hyperplasia in FVB/N mice. Comparative medicine. 2003;53(4):433-8.

14.	Radaelli E, Arnold A, Papanikolaou A, Garcia-Fernandez RA, Mattiello S, Scanziani E, et al. Mammary tumor phenotypes in wild-type aging female FVB/N mice with pituitary prolactinomas. Veterinary pathology. 2009;46(4):736-45.

**Supplementary Data**

**Supplementary Table 1: Antibodies used in study.**

| **Antibody** | **Manufacturer** |  | **Application** |  |
| --- | --- | --- | --- | --- |
| **GSK-3 (Total)** | Life Technologies |  | Immunoblot (IB) |  |
| **GSK-3β** | Cell Signaling Technology 9315 |  | Immunohistochemistry (IHC) |  |
| **β-catenin** | BD Transduction (mouse) |  | IB, IHC |  |
| **β-catenin** | Cell Signaling Technology 9581 (Rabbit) |  | IHC |  |
| **Keratin 8/18** | Fitzgerald |  | IHC |  |
| **Keratin 14** | Covance |  | IHC, Immunofluorescence (IF) |  |
| **Keratin 6** | Covance |  | IHC |  |
| **α-Smooth Muscle Actin** | Novus Biologics |  | IHC |  |
| **E-cadherin** | BD Transduction |  | IHC |  |
| **Ki67** | Biocare Medical |  | IHC |  |
| **Cyclin D1** | Calbiochem |  | IB |  |
| **BrdU** | abcam |  | IHC |  |
| **Cre** | Covance |  | IHC, IB |  |
| **EGFR** | Cell Signaling Technology 2646 |  | IHC |  |
| **γ-catenin** | Sigma clone 15F11, P8087 |  | IB |  |

**Supplementary Table 2: Genotyping PCR primers.**

| **Primer** | **Sequence** |
| --- | --- |
| **GSK-3α** | S: CCCCCACCAAGTGATTTCACTGCTA |
|  | AS: AACATGAAATTCCGGGCTCCAACTCTAT |
| **GSK-3β** | S: GGGGCAACCTTAATTTCATT |
|  | AS: TCTGGGCTATAGCTATCTAGTAACG  Exon: GTGTCTGTATAACTGACTTCCTGTGGC |
| **β-catenin exon 3** | S: GACACCGCTGCGTGGACAATG |
|  | AS: GTGGCTGACAGCAGCTTTTCTG |
| **β-catenin exon 2-6** | S: AAGGTAGAGTGATGAAAGTTGTT |
|  | AS: CACCATGTCCTCTGTCTATTC  Exon: TACACTATTGAATCACAGGGACTT |
| **Cre** | S: GTGCAAGTTGAATAACCGGAAATGG |
|  | AS: AGAGTCATCCTTAGCGCCGTAAATCA |
